# Co-Crystalization and *In Vitro* Biological Characterization of 5-Aryl-4-(5-Substituted-2-4-Dihydroxyphenyl)-1,2,3-Thiadiazole Hsp90 Inhibitors

**DOI:** 10.1371/journal.pone.0044642

**Published:** 2012-09-11

**Authors:** Swee Y. Sharp, S. Mark Roe, Egidijus Kazlauskas, Inga Čikotienė, Paul Workman, Daumantas Matulis, Chrisostomos Prodromou

**Affiliations:** 1 Cancer Research UK Cancer Therapeutics Unit, Division of Cancer Therapeutics, The Institute of Cancer Research, Haddow Laboratories, Sutton, Surrey, United Kingdom; 2 Biochemistry and Molecular Biology, Chichester 2, University of Sussex, Brighton, Falmer, United Kingdom; 3 Laboratory of Biothermodynamics and Drug Design, Institute of Biotechnology, Vilnius University, Vilnius, Lithuania; 4 Department of Organic Chemistry, Faculty of Chemistry, Vilnius University, Vilnius, Lithuania; 5 Genome Damage and Stability Centre, University of Sussex, Brighton, Falmer, United Kingdom; University of Washington, United States of America

## Abstract

A potential therapeutic strategy for targeting cancer that has gained much interest is the inhibition of the ATP binding and ATPase activity of the molecular chaperone Hsp90. We have determined the structure of the human Hsp90α N-terminal domain in complex with a series of 5-aryl-4-(5-substituted-2-4-dihydroxyphenyl)-1,2,3-thiadiazoles. The structures provide the molecular details for the activity of these inhibitors. One of these inhibitors, ICPD 34, causes a structural change that affects a mobile loop, which adopts a conformation similar to that seen in complexes with ADP, rather than the conformation generally seen with the pyrazole/isoxazole-resorcinol class of inhibitors. Competitive binding to the Hsp90 N-terminal domain was observed in a biochemical assay, and these compounds showed antiproliferative activity and induced apoptosis in the HCT116 human colon cancer cell line. These inhibitors also caused induction of the heat shock response with the upregulation of Hsp72 and Hsp27 protein expression and the depletion of Hsp90 clients, CRAF, ERBB2 and CDK4, thus confirming that antiproliferative activity was through the inhibition of Hsp90. The presence of increased levels of the cleavage product of PARP indicated apoptosis in response to Hsp90 inhibitors. This work provides a framework for the further optimization of thiadiazole inhibitors of Hsp90. Importantly, we demonstrate that the thiadiazole inhibitors display a more limited core set of interactions relative to the clinical trial candidate NVP-AUY922, and consequently may be less susceptible to resistance derived through mutations in Hsp90.

## Introduction

The molecular chaperone Hsp90 is responsible for the maturation and activation of specific client proteins that are key components of signal-transduction pathways that regulate growth and proliferation. These clients include numerous oncogenic proteins such as steroid-hormone receptors and kinases (ERBB2, EGFR, ALK, CRAF, BRAF and CDK4). The ATPase activity of Hsp90 is crucial for the activation of such client proteins. ATP binding to the N-terminal domain of Hsp90 leads to a series of structural changes that promote N-terminal dimerization [1], while binding Hsp90 inhibitors that target the ATP binding site of Hsp90 prevents these conformational changes and leads to the degradation of its client proteins [2].

The natural antibiotic Hsp90-inhibitors, geldanamycin and radicicol, target the N-terminal ATP-binding site of Hsp90. Inhibition elicits proteosomal degradation of Hsp90 client-proteins by a ubiquitination-mediated process, which may involve the E3 ubiquitin ligase CHIP [3]. Radicicol has no activity in vivo due to its instability and geldanamycin displays significant toxicity that precludes its use as an effective anticancer drug. This led to the development of the geldanamycin derivative 17-allylamino-17-demethoxy-geldanamycin (17-AAG, tanespimycin) [4], [5], [6], which has shown clinical activity in phase I/II clinical trials [7], [8], [9], [10]. Despite its clinical activity, most promisingly in trastuzumab-refractory ErbB2-positive breast cancer [10], 17-AAG suffers from a limited aqueous solubility, low oral bioavailability [10], [11], susceptibility to the metabolic activities of polymorphic enzymes (CYP3A4 and NQO1/DT-diaphorase [5], [12], [13]), and hepatotoxicity [7], [8], [9]. More water-soluble derivatives of geldanamycin, 17-DMAG (alvespimycin) and IPI-504 (retaspimycin), have entered clinical trials [10], [14], [15], [16]. Currently, radicicol derivatives have not entered clinical trial.

**Figure 1:**
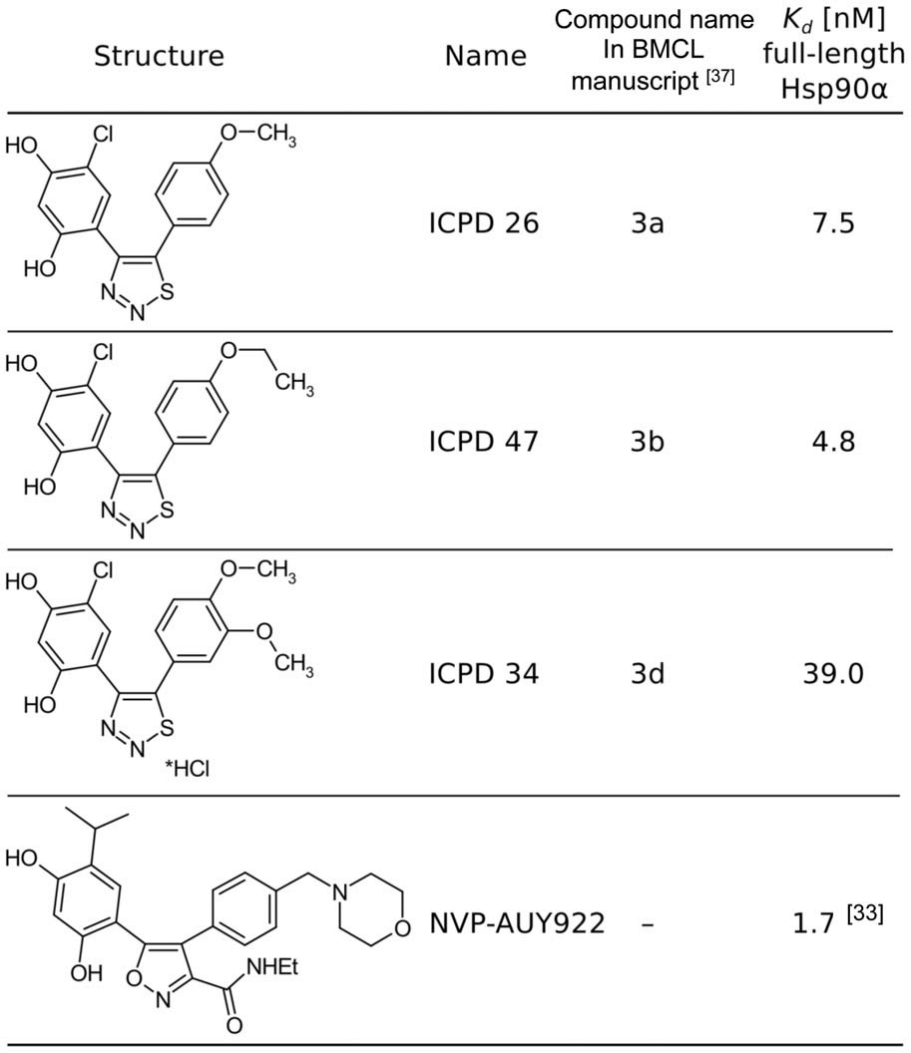
Chemical strucures of the thiadiazole compounds and NVP-AUY922. The K_d_ values for binding to Hsp90 are indicated.

Cancer cells appear to be more susceptible to Hsp90 inhibition than normal cells [17], [18], [19], [20], [21], [22] and consequently there have been considerable efforts to develop synthetic small molecule inhibitors against the ATP-binding site of Hsp90 [23], [24]. The first synthetic small molecule to be identified as a Hsp90 ATPase-inhibitor was based on a purine scaffold [25], [26]. Another class of small molecules, the 3-4-diaryl pyrazole resorcinols, was then identified. The pyrazoles are exemplified by the prototype CCT018159 [27], [28], [29], and were further optimized to produce the pyrazole- and isoxazole-amide resorcinol analogues [30], [31], from which the isoxazole NVP-AUY922 (VER52296, Fig. 1) emerged as a clinical trial candidate that is now showing promise in Phase II clinical trials [32], [33], [34]. These new agents overcome many of the liabilities of the geldanamycin class, including hepatotoxicity that could be attributed to the quinone group [23], [24].

While mechanisms of resistance to Hsp90 inhibitors have so far not emerged in the clinic, it has been clearly demonstrated that resistance to the natural product inhibitors, geldanamycin and radicicol, is possible through mutation leading to altered amino-acid residues in the ATP-binding site of Hsp90 [35], [36]. Thus it appears that the ATP-binding pocket of Hsp90, although highly conserved, can nevertheless tolerate mutagenic changes leading to resistance against these inhibitors. Potentially, such mutations may eventually be seen in the clinic and consequently the development of a variety of structurally diverse inhibitors, that interact purely with highly conserved residues that form the central components of the ATP-binding site of Hsp90, is all the more important. The present series of the 5-aryl-4-(5-substituted-2-4-dihydroxyphenyl)-1,2,3-thiadiazoles (ICPD 26, 34 and 47) were recently synthesized (Fig. 1) and shown to be effective Hsp90 inhibitors in terms of binding to Hsp90 [37]. The dissociation constant for the binding of these inhibitors to full-length Hsp90α varied from 4.8 to 39.0 nM. Here we determine the molecular and structural biological details of this inhibition, demonstrate using biomarkers that these agents inhibit Hsp90 in cancer cells, and show that they exhibit antiproliferative activity and induce apoptosis in the human colon cancer cell line HCT116. Importantly, we demonstrate that the thiadiazole inhibitors display a more limited core set of interactions relative to the clinical trial candidate NVP-AUY922, and consequently may be less susceptible to resistance derived through mutations in the N-terminal ATP site of Hsp90.

## Results

### Protein X-ray Crystallography

**Figure 2:**
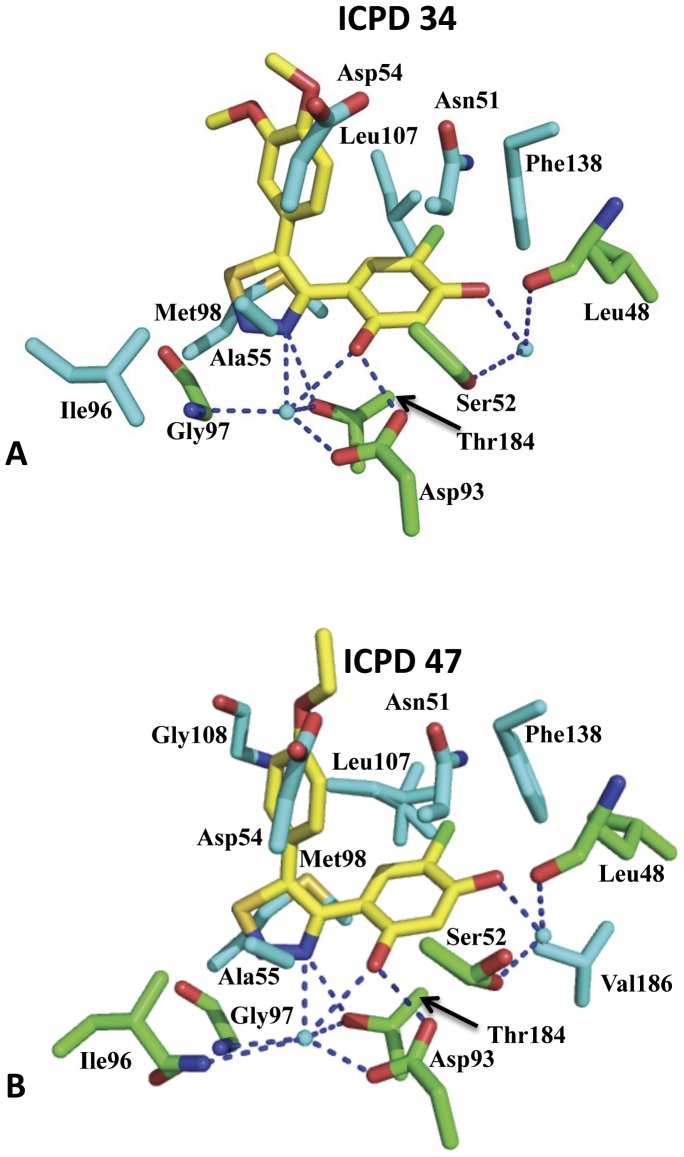
PyMOL diagram showing binding interactions with Hsp90. (**A**) Interactions of ICPD 34 and (**B**) ICPD 47 with the N-terminal domain of human Hsp90α. Dotted blue lines represent hydrogen bonds and the amino-acid residues involved are in green; cyan-colored spheres represent water molecules and cyan residues are amino acids solely in van der Waals contact. The structures for ICPD 34, and ICPD 47 were obtained at 2.5, and 1.4 Å resolution, respectively. ICPD 26, which lacks a 3-methoxy group relative to ICPD 34, binds in essentially the same manor as ICPD 34 and ICPD 47. For atomic coordinates and structure factors, see PDB codes 2YI0 (ICPD 26), 2YI5 (ICPD 34), and 2YI7 (ICPD 47).

ICPD 26, 34 and 47 (Fig. 1) essentially bind to Hsp90 in the same way (Fig. 2 and Table 1). A crucial network of hydrogen bonding between the resorcinol group and highly conserved amino-acid residues, Leu 48, Ser 52, Asp 93, Gly 97 and Thr 184, of the ATP-binding site is present as previously seen with pyrazole and isoxazole resorcinol inhibitors [27], [28], [32] (Fig. 2). Hydrophobic interactions that are also conserved include those with Asn 51, Ala 55, Met 98 and Phe 138 (Fig. 2). The interaction between Gly 97 and the monoethylamide group of NVP-AUY922 has previously shown to be important for the potency of this compound [21], [31]. The main-chain carbonyl group of Gly 97 does not form a hydrogen bond interaction with the ICPD compounds (Fig. 2). Furthermore, the sulphur atom in the thiadiazole ring of the ICPD compounds is not optimally positioned to form an interaction with the carbonyl of Gly 197 (Fig. 2). The chlorine atom in ICPD 34 and ICPD 47 and the interactions it makes has been previously explored with other compounds containing chlorine at the same position. The interactions made by the chlorine atom are consistent with these reports [27], [28], [32].

**Table 1:** Crystallographic statistics.

Data Set(Highest shellin parentheses)	ICPD 26	ICPD 34	ICPD 47
a (Å)	65.430	70.870	65.230
b (Å)	89.315	90.310	88.705
c (Å)	100.090	88.440	99.625
α (°)	90.00	90.00	90.00
β (°)	90.00	90.00	90.00
γ (°)	90.00	90.00	90.00
Space Group	I222	C222_1_	I222
Wavelength (Å)	0.97960	0.97960	0.97960
Resolution Limit (Å)	53.0–1.6(1.69–1.60)	56.0–2.50(2.63–2.50)	45.0–1.40(1.48–1.40)
Number of Obs.	38928 (5611)	10079 (1447)	57073 (8252)
Completeness (%)	99.8 (99.9)	99.6 (99.6)	99.8 (100.0)
Multiplicity	4.1 (3.8)	3.4 (3.5)	4.2 (3.9)
Rmrg (%)	0.057 (0.317)	0.083 (0.524)	0.047 (0.392)
I/σI	16.0 (4.0)	12.2 (2.3)	18.3 (3.3)
Refinement			
R_cryst_	0.199	0.2042	0.203
R_free_	0.234	0.2589	0.229
No. of protein atoms	1639	1616	1658
No. of ligand atoms	23	24	24
No. of solvent atoms	303	107	366
Mean B	18.6	43.2	14.0
Rmsd bond lengths (Å)	0.020	0.020	0.017
Rmsd bond angles (°)	1.946	0.713	1.868

**Figure 3:**
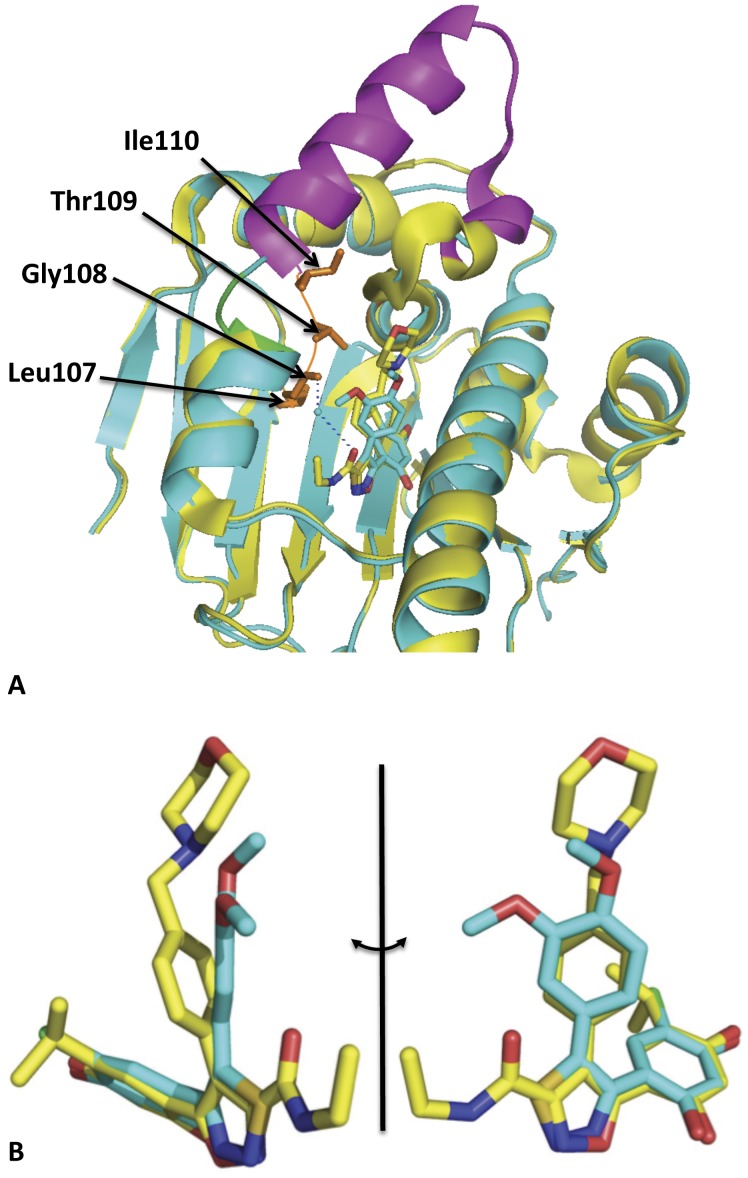
PyMol cartoons of the structure of human Hsp90-drug complexes. (**A**), PyMol cartoon showing ICPD 34 (cyan) and NVP-AUY922 (yellow and magenta) bound to the N-terminal domain of human Hsp90α. The magenta region of the NVP-AUY922-Hsp90α complex represents the unstructured region of the ICPD 34-Hsp90α complex. The mobile loop region interacting with NVP-AUY922 is shown in gold. Water molecules are shown as cyan colored spheres and hydrogen bonds as dotted blue lines. The 3-methoxy group of ICPD 34 causes the loop represented by Asn 105 to Ile 110 to adopt an alternative conformation, which moves from the gold to green conformation. (**B**), Two orthogonal views of the superimposition of ICPD 34 (Cyan) and NVP-AUY922 (yellow) showing that the compounds bind with overall similarity, except for the morpholino group of NVP-AUY922 and the dimethoxyphenyl group of ICPD 34. The structures for ICPD 34 and NVP-AUY922 were obtained at 2.5 and 2.0 Å resolution, respectively. For atomic coordinates and structure factors, see PDB codes 2YI5 and 2VCI, respectively.

Although the ICPD compounds bind Hsp90 in similar ways, the K_d_ values for the binding of ICPD 26 and ICPD 47 (7.5 and 4.8 nM, respectively) and ICPD 34 (K_d_ = 39.0 nM) are very different, with ICPD 34 being less potent (Fig. 1). ICPD 26 is similar to ICPD 34, except that it lacks a 3-methoxy group (Fig. 1 and 2). The presence of this group in ICPD 34 has a profound effect on a short mobile loop (Asp 105 to Ile 110) that consequently adopts an open conformation and fails to interact with the bound inhibitor (Fig. 3A). Furthermore, amino-acid residues 111–131 become completely disordered (Fig. 3A). With ICPD 26 and 47 this loop adopts a more compact conformation interacting with the bound inhibitor. For a variety of pyrazole and isoxazole inhibitors the conformation of the loop is either in an open (pdb 2BYI and 2BYH) or compact conformation (pdb 2CCU, 2VCJ, 2CCT, 2CCS, 2BSM and 2BT0). For the clinical trial candidate NVP-AUY922 (pdb 2VCI) the loop is in the apparent favored compact state. It therefore appears that the binding affinity of ICPD 34 is negatively affected by having to promote a conformational change that moves the short mobile loop to a more open conformation and causes disorder in the amino-acid residues from Asp 105 to Ile 110, and/or by failing to interact with the loop itself. This could potentially explain the weaker binding of ICPD 34, relative to ICPD 26 and 47 (Fig. 1–3). Consequently, optimizing interactions between this mobile loop and future Hsp90 inhibitors could provide higher affinity binding inhibitors as has been observed for the morpholino- and monoethylamide-group of NVP-AUY922 (pdb 2VCI). Such interactions should be directed towards amino acid residues closest to the bound inhibitors, which include the highly conserved Leu 107, Gly 108 and Thr 109 amino-acid residues. However, because these residues are found at the periphery of the ATP-binding site they may be subject to mutational changes that could result in resistance.

Resistance through mutagenesis of the ATP-binding site of Hsp90 has recently been shown against both radicicol and geldanamycin [35], [36]. Interestingly, resistance to geldanamycin involved amino acid changes (E88G and N92L) that are at the entrance to the ATP-binding site of Hsp90. In contrast, resistance to radicicol involved an amino acid change (L34I) at the base of the ATP-binding pocket. However, because of the less bulky nature of the thiadiazoles and the simpler set of interactions that these compounds make with Hsp90, they have the potential to be potent inhibitors in situations where more bulky compounds, such as NVP-AUY922, may fail due to emerging resistance in Hsp90. Together with their significant activity against Hsp90, the structural data indicate that the thiadiazole inhibitors may represent a very useful class of Hsp90 inhibitor.

### Thiadiazoles Bind to Hsp90β and Inhibit Proliferation of HCT116 Human Colon Cells

The thiadiazoles compounds were evaluated for binding to full-length human Hsp90β and antiproliferative activity in cancer cells using the fluorescence polarization (FP) and the sulforhodamine B (SRB) assay, respectively (Table 2). Competitive binding as measured by FP across the 3 compounds ranged from 14.6 to 51.9 nM, which were consistent with the measured K_d_ values (Fig. 1). The compounds exhibited antiproliferative activity (GI_50_ = 3.2 to 4.6 µM) against the HCT116 human colon cancer cell lines (Table 2).

**Table 2:** Hsp90 binding and inhibitory activities.

Compound	FP (IC_50_; nM)	HCT116 SRB (GI_50_; µM)
ICPD 26	26.6±4.4	3.2±0.7
ICPD 34	51.9±6.4	4.6±0.4
ICPD 47	14.6±1.6	4.6±0.4

FP and SRB values are mean ± SE of assays done in triplicate.

### Thiadiazoles Elicit Depletion of Hsp90 Client Proteins, Induction of Heat Shock Proteins and Apoptosis

**Figure 4:**
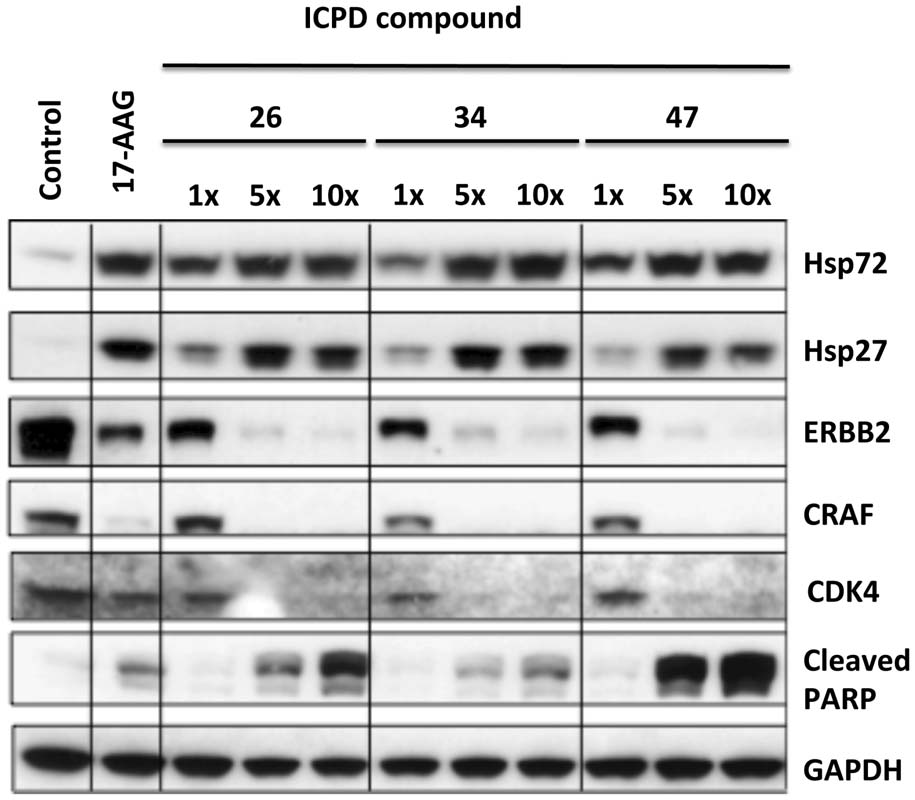
The biomarker signature of Hsp90 inhibition. Western blot showing depletion of Hsp90 client proteins and induction of Hsp72, Hsp27 and cleaved PARP, upon treatment with 1×, 5× and 10× GI_50_ concentrations of compounds in HCT116 human colon cancer cells for 24 hr. 17-AAG was used as a positive control. Gapdh was used as loading control. Induction of cleaved PARP is also shown and is indicative of apoptosis.

To confirm that growth inhibition was due to the intended Hsp90 inhibitory mechanism, the ICPD 26, 34 and 47 compounds were assessed for their effects on the established molecular signature of Hsp90 inhibition in the HCT116 cancer cells [38]. Depletion of client proteins CRAF, ERBB2 and CDK4, together with upregulation of Hsp72 and Hsp27 expression was observed, with a series of inhibitor concentrations, for the three compounds currently available, ICPD 26, 34 and 47, thus confirming that these compounds were acting as Hsp90 inhibitors in the cell (Fig. 4). An increase in PARP cleavage indicated induction of apoptosis.

## Discussion

The thiadiazoles studied here were previously shown to have significant affinity towards Hsp90, displaying K_d_ values ranging from 4.8 and 39 nM [37]. Herein we have characterized the molecular interactions that these inhibitors make with the Hsp90α N-terminal ATP-binding domain and analyzed their effects on the human colon cancer cell line HCT116. The X-ray structures presented herein demonstrate that the thiadiazole compounds, although chemically simpler and less bulky, are none-the-less effective Hsp90 inhibitors. One of the inhibitors, ICPD 34, causes a structural change that affects a mobile loop, which adopts a conformation similar to that seen in complexes with ADP, rather than the conformation generally seen with the pyrazole/isoxazole-resorcinol class of inhibitors, such as NVP-AUY922. Importantly, we demonstrate that the thiadiazole inhibitors display a more limited core set of interactions relative to the clinical trial candidate NVP-AUY922, and consequently may be less susceptible to resistance derived through mutations in Hsp90. We have not tested this possibility directly, as it is difficult to predict precisely what mutations in Hsp90 might arise that would lead to resistance. However, the possibility of avoiding resistance combined with their antiproliferative and apopototic activity mediated through Hsp90 inhibition, makes the thiadiazole compounds an interesting and potentially useful class of Hsp90 inhibitors.

Interestingly, a conformational change in the main-chain of the N-terminal domain was seen with ICPD 34, in order to accommodate the 3-methoxy group on the benzyl ring of this compound. It appears that the resulting conformation of the loop upon binding of ICPD 34 lowers the binding affinity as compared with ICPD 26 and ICPD 47, which lack the 3-methoxy group. The additional interactions this group makes do not compensate for the loss of the morpholino group, which for NVP-AUY922 is involved in a series of interactions that contribute to the increased potency of this compound [33].

The IC_50_ values for binding of the clinical candidate NVP-AUY922 to Hsp90α and Hsp90β, as measured by the FP competitive binding assay, were found to be 7.8±1.8 and 21±16 nM, respectively [33]. In comparison, the thiadiazoles displayed values ranging from 14.6 to 51.9 nM against Hsp90β, results that are consistent with their somewhat weaker affinity for Hsp90 relative to NVP-AUY922. Furthermore, the GI_50_ values for antiproliferative activity ranged between 3.2 to 4.6 µM, which are much higher than the values for NVP-AUY922 (16.0±8.0 nM) against the HCT116 human colon cancer cell line [33]. The molecular signature of Hsp90 inhibition is the induction of the molecular chaperones Hsp72 and Hsp27 combined with the depletion of Hsp90 client-proteins. All three of the thiadiazole compounds tested displayed this signature of Hsp90 inhibition (Fig. 4) and they also induced PARP cleavage, suggesting that they induced apoptosis. The greater cellular potency of NVP-AUY922 against the thiadiazoles may be due to cell permeability issues and it is acknowledged that further optimization work is required to convert the high potency on the molecular target into greater potency against cancer cells. Such investment of effort can be justified by their potential to avoid emerging mechanisms of resistance due to mutations in the N-terminal ATP pocket.

In conclusion, apart from the relative simplicity of their synthesis, the main potential advantage of the thiadiazole compounds studied here is the limited number of interactions that they make with highly conserved amino-acid residues representing the core of the N-terminal ATP-binding site of Hsp90. Consequently, they maybe less prone to resistance through mutation of this ATP-binding site. Whether these compounds could be developed further remains to be seen; however, this present study provides the structural basis and mechanistic framework to support further optimization.

## Materials and Methods

### Hsp90 Inhibitors

The synthesis of the 5-aryl-4-(5-substituted-2-4-dihydroxyphenyl)-1,2,3-thiadiazoles was previously described [37]. The chemical structures of the compounds studied here are shown in Figure 1.

### Crystallization, Crystallographic Data Collection and Refinement

Crystals of human Hsp90α N-terminus were grown from a 1:1 mixture of protein (29 mg ml^−1^) and precipitant solution (0.2 M MgCl2, 25% PEG 2 K and 100 mM sodium cocodylate pH 6.5) using the vapor diffusion crystallization method. The crystals were stepwise cryoprotected in 30% glycerol and flash frozen before data were collected at Diamond on beamline IO2. Data were processed in Mosflm and scaled and merged using SCALA [39]. The structure was solved by molecular replacement with Phaser using pdb code 2WI1 for compounds ICPD 26, and 47, and PDB code 1YC1 for ICPD 34 as the search model. The structures were refined using Phenix, with weight optimization, and manual building using COOT [40], [41], [42]. The inhibitors were built using the PRODRG^5^ server. The ligand and waters were added towards the end of refinement. Structures were submitted to PUBSUM [43] for determination of molecular interactions between the protein and the drug molecules, in addition to visual inspection with PyMol [44]. The statistics for the crystallographic determinations are shown in Table 1 and the co-ordinates for the structures were deposited in the pdb database. The pdb accession numbers are 2YI0 (ICPD 26), 2YI5 (ICPD 34), and 2YI7 (ICPD47).

### Cell Lines

The human colon cancer cell line HCT116 was obtained from the American Type Culture Collection (LGC Promochem, Middlesex, UK). Cells were grown in DMEM/10% FCS, 2 mM glutamine and nonessential amino acids in 5% CO_2_. HCT116 cells were free of Mycoplasma contamination (Venor GeM kit, Minerva Biolabs, Berlin, Germany).

### Production of Recombinant Proteins

The N-terminal domain of Hsp90α [45] and the full length Hsp90β proteins were overexpressed in E. coli and purified as previously described [46], [47].

### Western Blots

Protein extracts were prepared and Western blotting was performed as previously described [5], [31].

### Fluorescence Polarization and Growth Inhibition Assay

Binding of HSP90 inhibitors to human full-length HSP90β was determined by a competitive binding fluorescence polarization assay, using a fluorescent isoxazole- resorcinol probe, as described previously [48]. Briefly, compounds were assayed in a 96-well plate format to determine IC_50_ values. Reaction mix (100 µl; 100 mM Tris.Cl, pH 7.4, 20 mM KCl, 6 mM MgCl2, 5 µg ml^−1^ bovine serum albumin, 80 nM isoxazole probe, 100 nM Hsp90β) was added per well of a black 96-well plate (Corning Costar No. 3915) and allowed to equilibrate for 20 min at room temperature in the dark. Plates were read on a Fusion Alpha-FP (Perkin–Elmer, USA) with excitation 485/20 nM and emission 535/25 nM with polarization. Compounds at a range of concentration were then added to the reaction, mixed thoroughly, and allowed to equilibrate again at room temperature in the dark for 20 min. IC50 values were calculated on the difference in anisotropy from the first and second reads.

Sensitivity of cells to Hsp90 inhibitors was measured by the sulforhodamine B (SRB) assay [5]. Briefly, cells were seeded into 96-well microtiter plates and allowed to attach for 36 hours. Compounds at a range of concentrations were added in quadruplicate wells for 4 days in a volume of 200 µl per well. The GI_50_ was calculated as the drug concentration that inhibits cell proliferation by 50% compared with vehicle controls.
